# Efficacy of a Biocidal Paint in Controlling *Alphitobius diaperinus* (Panzer) (Coleoptera: Tenebrionidae) and Improving the Quality of Air and Litter in Poultry Houses

**DOI:** 10.3390/ani12101264

**Published:** 2022-05-14

**Authors:** Sara Dzik, Tomasz Mituniewicz, Ariphzan Beisenov

**Affiliations:** 1 Department of Animal and Environmental Hygiene, University of Warmia and Mazury in Olsztyn, 5 Oczapowski Street, 10-719 Olsztyn, Poland; t.mituniewicz@uwm.edu.pl; 2 Department of Technology and Biological Resources, Kazakh National Agrarian Research University, 8 Abai Avenue, Almaty 050010, Kazakhstan; aripzan@mail.ru

**Keywords:** broiler houses, farm hygiene, disinfection, disinsection, lesser mealworm, insect pest, microbiological contamination, microclimate conditions, poultry litter

## Abstract

**Simple Summary:**

Poultry meat production and consumption continue to grow worldwide; therefore, the safety of poultry products is a priority for consumers, producers, and governing authorities. Thus, it is essential to find an effective solution to maintain proper hygiene in poultry production. One such tool is a water-based slow-release biocidal paint that contains insecticidal and antimicrobial compounds. In this study it was assumed that biocidal paint could be useful in controlling insect pests such as *Alphitobius diaperinus* and reducing microbiological contamination of air and litter and have a beneficial effect on microclimate in poultry houses. The results suggest that the tested biocidal paint could be an effective alternative to other insecticides and disinfectants. Additionally, the research is of a practical nature and may be very useful for poultry producers in controlling *A. diaperinus* populations and maintaining proper hygiene in poultry houses.

**Abstract:**

Effective disinfection and disinsection are the keys to successful operation of modern poultry farms and the safety of poultry products. The cleaning and disinfection of poultry houses are important aspects of farm hygiene management. The correct execution of all steps of cleaning, disinfection, and disinsection procedures and the use of appropriate products are crucial for the prevention and control of zoonotic and animal diseases. In this study it was assumed that a water-based slow-release biocidal paint could be useful in controlling insect pests such as *Alphitobius diaperinus* and reducing microbiological contamination of air and litter in poultry houses and have a beneficial effect on microclimate in poultry houses. Therefore, the locations of *A. diaperinus* in the poultry houses, the microbiological contamination of air and litter, as well as the microclimatic conditions in the houses and the physicochemical parameters of the litter were evaluated. The results suggest that the tested biocidal paint could be an effective alternative to other insecticides and disinfectants. Additionally, the research is of a practical nature and may be very useful for poultry producers in controlling *A. diaperinus* populations and maintaining proper hygiene in poultry houses. Further research is needed.

## 1. Introduction

*Alphitobius diaperinus* (Panzer) (Coleoptera: Tenebrionidae), also known as the lesser mealworm, is an insect able to live in human-transformed environments, mainly on poultry farms [1,2,3,4], especially when chickens are kept on litter [5]. The high air temperature in poultry houses and a layer of moist litter promote beetle development throughout the year [6]. In addition, Voris et al. [7] noted the seasonality of *A. diaperinus* whose abundance increased with a rise in outdoor air temperature.

In poultry houses, *A. diaperinus* may feed on dead and diseased birds, chicken manure, and other organic materials. The insect is a vector and a reservoir of many poultry pathogens [8,9], including bacteria (e.g., *Salmonella* spp., *Campylobacter* spp., *Staphylococcus* spp., *Escherichia coli*), fungi (*Aspergillus* spp., *Candida* spp.), protozoa (*Eimeria* spp.), and other parasites of poultry, such as *Hymenolepis nana*, as well as viruses (causing Marek’s disease, Gumboro disease, Newcastle disease, avian influenza) [10]. Apart from the direct effects mentioned above, *A. diaperinus* has also been reported to cause serious damage to poultry facilities and building structures, thus generating economic losses [1,8].

Both high stocking density and intensive farming may lead to the development of pathogenic microorganisms in poultry houses, thus increasing the risk of infectious diseases [11,12]. The “from farm to fork” strategy emphasizes the importance of monitoring the safety of food products already at the primary production stage [13]. Poultry meat production and consumption continue to grow worldwide; therefore, the safety of poultry products is a priority for consumers, producers, and governing authorities [14]. Poultry meat has been identified as one of the most important pathogen carriers [14,15,16].

In intensive livestock production areas, air is contaminated with high concentrations of microorganisms, mostly bacteria [17,18,19]. Bacterial aerosols consist mainly of staphylococci and streptococci as well as numerous Gram-positive and Gram-negative bacteria [18,20,21]. The microclimate in poultry facilities is determined by the physicochemical properties of litter. Factors such as temperature, humidity, and litter pH influence the abundance and activity of microorganisms, both in the litter itself and in the air in livestock buildings [22,23,24,25]. Broiler chickens on poultry farms usually remain in contact with litter throughout the rearing period; therefore, appropriate litter quality is an important consideration [26]. Various factors, such as improper litter handling, inadequate room ventilation, as well as excessive litter moisture, can deteriorate litter quality [27]. In addition, high litter pH can significantly accelerate the decomposition of uric acid to ammonia [28].

In view of environmental and epidemiological risks, continuous monitoring and control of microorganisms and their vectors in the poultry house environment are important considerations. Therefore, effective disinfection [12] and disinsection [29] are the keys to successful operation of modern poultry farms and the safety of poultry products. The cleaning and disinfection of poultry houses are important aspects of farm hygiene management. The correct execution of all steps of cleaning, disinfection, and disinsection procedures and the use of appropriate products are crucial for the prevention and control of zoonotic and animal diseases [29,30].

It is important to find an effective solution to maintain proper hygiene in poultry production. One of such tools is a slow-release water-based biocidal paint [29] that contains insecticidal (a chemical insecticide and an optical repellent against *A. diaperinus*) and antimicrobial compounds. The chemical insecticide is permethrin, a synthetic pyrethroid that can be used against several insect species [31,32,33,34]. Permethrin acts as a stomach poison when eaten by insects or as a contact poison through direct contact with target pests. If an insect consumes or has direct contact with permethrin, it affects its nervous system. It is effective against eggs, larvae, pupae, and adults [35]. Moreover, pyrethroids are recommended for use against insects (unless resistance has been confirmed) due to their rapid destruction and high insecticidal efficacy at low doses [36,37]. The optical repellent is a mixture of ultramarine and violet 23 [34]. Light sensing is critical for insects [38]. Many beetles are vulnerable to blue and violet light (sensitive photoreceptors) [39,40]. Thus, the addition of a blue pigment (ultramarine) and a violet pigment (violet 23) to the paint may provide additional protection against *A. diaperinus*. Various paints with insecticidal properties are known, but they have not been used in poultry houses against *A. diaperinus* to date. 

The antimicrobial active ingredients of the tested biocidal paint are zinc pyrithione and 1,2-benzisothiazol-3(2H)-one. In contrast to other antimicrobial ingredients with silver and copper, zinc-based ones are broad-spectrum antimicrobial agents [41,42,43]. Zinc pyrithione inhibits fungal growth through copper influx and damage to iron-sulfur proteins; it also depolarizes membrane electropotential in fungi [44,45]. 1,2-benzisothiazol-3(2H)-one is also known as an effective antimicrobial agent [46,47,48].

Dzik and Mituniewicz [29] demonstrated that the analyzed biocidal paint was fully effective in laboratory conditions. When applied in poultry houses, it did not show 100% efficacy against *A. diaperinus* but was more effective than liming. In this study, the assumption was made that insect pests would be found in traps placed farther away from the walls of a house where the tested biocidal paint was applied, pointing to its repellent properties. It was also assumed that the application of the biocidal paint (antimicrobial ingredients), as compared with liming, would reduce microbial contamination of the air and litter as well as improve microclimate conditions in the house and the physicochemical parameters of the litter.

## 2. Materials and Methods

The experiment involved a routine hygiene procedure carried out on a production farm. Therefore, according to Polish law, the consent of the Local Ethics Committee for Experiments on Animals was not required, as no treatment, including medical treatment, invasive diagnostics, or procedures causing psychological or social discomfort to the participants were used in this study. The experiment was conducted in accordance with European Union (EU) Directive 2010/63/EU. It was established on a poultry farm (Pomerania region, northern Poland) and included two groups. Each group comprised a poultry house with a maximum stocking density (20,500 Ross 308 chickens). Chickens were reared on rye straw litter (approx. 15 cm thick) for 40 days. Each rearing period was followed by a cleaning period of 3 weeks, after the birds had been removed. The houses (each with an area of 1365 m^2^—15 m × 91 m) were equipped with adjustable mechanical ventilation as well as artificial lighting with adjustable light intensity. In both poultry houses, birds had permanent access to drinking water and were fed standard commercial diets (Starter, 1–10 days; Grower I, 11–20 days; Grower II, 21–31 days; Finisher, 32–40 days, according to the manufacturer’s instructions, Schaap-Pol Ltd. Polczyno, Poland). 

The experimental factor was insecticide application. Lime (300 mL/m^2^) was applied in one poultry house, seven days before each production cycle, with a hydrodynamic aggregate (Faska Ltd., Fabianki, Poland). In the other poultry house, the interior (walls, floor, ceiling) was painted with a biocidal paint (Kleib Ltd., Brzesc Kujawski, Poland) applied at 400 mL/m^2^ with a hydrodynamic aggregate, at the beginning of the experiment (seven days before the first production cycle). The paint contains two active ingredients:

(i) chemical—permethrin (0.04% by weight),
(ii) optical—a mixture of ultramarine and violet 23 (0.004% by weight)

and two antimicrobial active ingredients:

(i) zinc pyrithione (0.12 % by weight),
(ii) 1,2-benzisothiazol-3(2H)-one (0.05% by weight).

Based on the method used, the groups were denoted as L (liming) and Bp (biocidal paint).

### 2.1. Location of A. diaperinus

**Figure 1:**
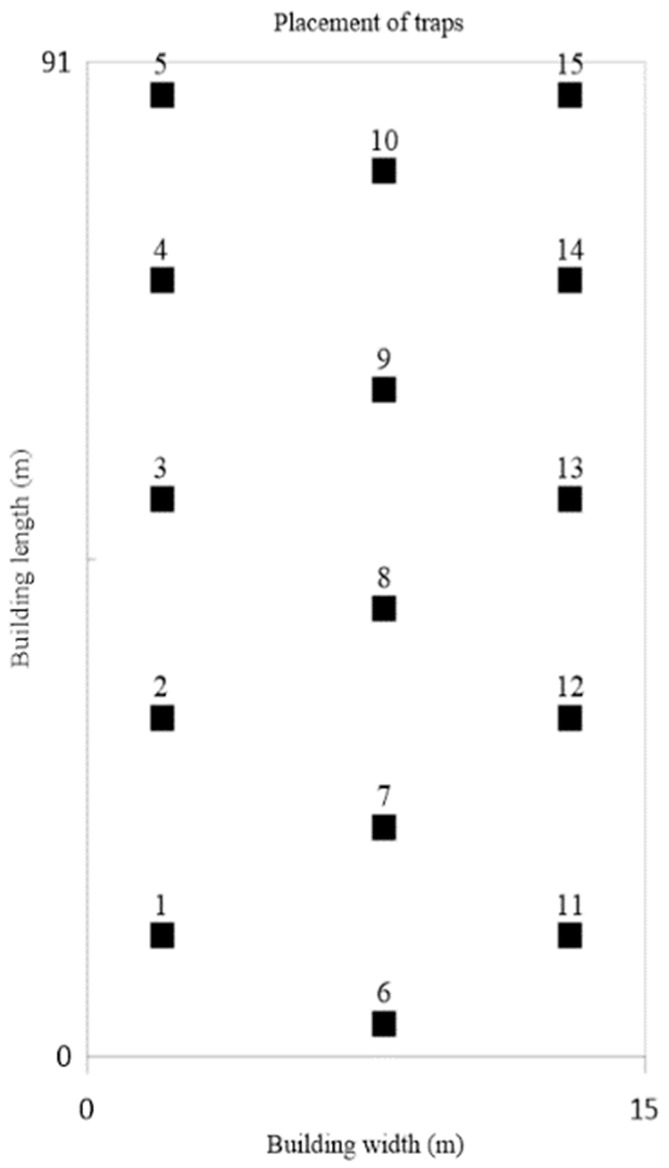
Setting up traps against *Alphitobius diaperinus* in poultry houses.

In order to assess the significance of spatial location, each house was divided into three sections, left (traps 1–5), middle (traps 6–10), and right (traps 11–15), in all trials. A total of 15 specialized traps [49] for trapping insect pests (“Tendrop”, Biomucha Company, Poznan, Poland) were used in each group. Traps were placed on the pavement, near the longitudinal walls of the house and in the middle of the house (between the feeder and drinker lines) (Figure 1). At the time of transition to new feed (days 10, 20, 31, and 40 of rearing), insects were collected from the traps, placed in plastic drawstring bags, and frozen for 48 h. Next, the insects were transferred to Petri dishes and counted.

### 2.2. Microbiological Contamination

Air samples for microbiological analyses were collected on days 1, 21, and 40 of rearing, by the air sedimentation method with the use of Petri dishes. In all trials, air sampling was performed in both poultry houses, at five measurement sites, in duplicate. Then, the total counts of mesophilic aerobes (EN-ISO 18593:2005) and fungi (EN-ISO 21528-2:2005) were determined under laboratory conditions. The bacteria present in Petri dishes were identified via macroscopic and microscopic methods using API biochemical tests and APIWEB software, (BioMèrieux Poland Ltd., Warsaw, Poland).

In all trials, the level of microbiological contamination of the walls was evaluated using the swabbing technique. Sterile templates measuring 10 cm × 10 cm and sterile cotton swabs moistened with sterile peptone saline were used to obtain samples from flat wall surfaces. In all trials, samples were collected on days 1, 21, and 40 of rearing, in both poultry houses, at four measurement sites, in duplicate. *Salmonella* spp. (EN-ISO 6579-1:2017) and other microorganisms were identified under laboratory conditions in two stages: (1) single colonies were isolated from plate cultures, (2) the microorganisms were identified to species level using API tests and APIWEB software.

In all trials, litter samples were collected for analyses on days 1, 21, and 40 of rearing, in both poultry houses, at eight measurement sites, in duplicate. Microbiological contamination was determined both quantitatively (the total counts of mesophilic aerobes: EN-ISO 18593:2005 and the total counts of fungi: EN-ISO 21528-2:2005) and qualitatively (identification of fungi: based on their morphological features with the use of identification keys [50,51]), under an MN-800 laboratory microscope (OPTA-TECH, Warsaw, Poland).

### 2.3. Microclimate Conditions and the Physicochemical Parameters of Litter

In all trials, temperature and relative air humidity were monitored in both poultry houses via continuous registration (every 15 min) using LB-520 thermo-hygrometers with an LB-521USB interface (LAB-EL, Reguly, Poland). Air NH_3_ concentration was measured with the DP-24 VET multi-gas detector (NANOSENS Ltd., Tarnowo Podgorne, Poland) at the height of birds’ heads, three times a day (at 15 sites near the insect traps). The results were presented for days 1, 10, 20, 31, and 40 of rearing.

The basic physicochemical parameters of litter were also evaluated: temperature and moisture of the surface layer of straw litter (to a depth of 5 cm) were measured with the HAYMETER HMM 1625/02 thermo-hygrometer (Draminski Inc., Olsztyn, Poland), NH_3_ levels were determined via the photometric method using the Ammonia PC Checkit (Lovibond, Dortmund, Germany), and litter pH was measured with an agricultural digital pH-meter, at 15 sites in each group (in each trap). The results were presented for days 1, 10, 20, 31, and 40 of rearing [23,24].

Throughout the experiment, seven production cycles were completed in the poultry houses (both groups). The experiment was repeated three times (three trials), during the first, third, and seventh production cycle, and each production cycle lasted 40 days (Table 1). There were no cases of infectious diseases on the farm during the experiment.

### 2.4. Statistical Analysis

Before statistical analyses, raw data were log-transformed. Thus, the distribution of data was approximated to normal with the Shapiro-Wilk test. In order to evaluate the effect of the tested biocidal paint on the number of insect pests found in traps placed in different sections of the house, repeated measures ANOVA (RMANOVA) was performed, where the disinfection and disinsection method used (liming or biocidal paint) and sections of the house (left, middle, right) were the fixed effects, and trials (1, 2, 3) as well as days of measurement (10, 20, 31, and 40) were the repeated measures factors. Tukey’s test (*p* < 0.05) was used to assess multiple comparisons and the significance of differences between treatment means. In the next step, the significance of differences in the number of *A. diaperinus* individuals trapped in different sections was assessed separately for each poultry house using orthogonal contrast analysis at a significance level of α = 0.05. Two contrasts (C#1 and C#2) were used to determine the differences between traps in the middle and outermost sections of the house, and between traps in the outermost sections (Table 2).

Traps with the highest and repeated numbers of *A. diaperinus* in all trials were selected and defined as “critical points”. Student’s *t*-test with *p* < 0.05 was used to determine the differences in the average numbers of pests trapped in selected “critical points” between two poultry houses on a given day of measurement.

The total counts of mesophilic aerobes and fungi in the air and litter (quantitative analysis) in the poultry houses were analyzed via RMANOVA followed by the Student’s *t*-test with *p* < 0.05.

Microclimate conditions and the physicochemical parameters of litter were compared between the groups using the Student’s *t*-test with *p* < 0.05, and multiple comparisons of the parameters within the groups were analyzed statistically using one-way RMANOVA followed by Tukey’s test (*p* < 0.05).

Statistical analyses were performed using Statistica v. 13.3.0 software [52].

## 3. Results

### 3.1. Location of A. diaperinus

**Figure 2:**
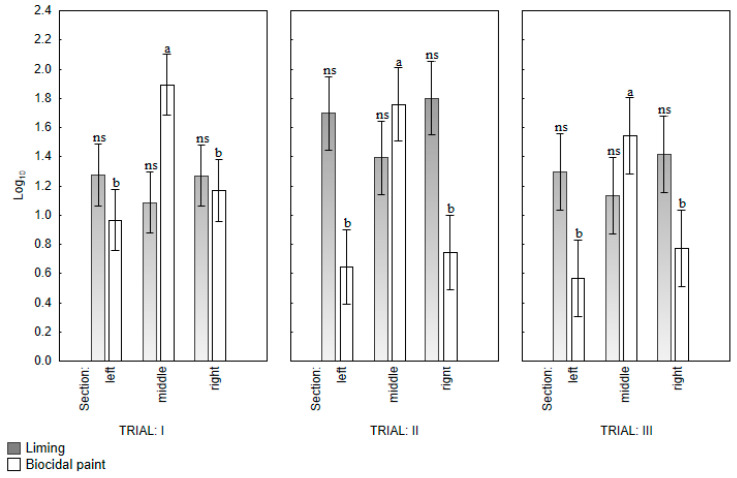
Location of *Alphitobius diaperinus* in different sections of poultry houses in each trial. Different lowercase letters indicate significant differences (*p* < 0.05); ns—no significant differences between trap sections. The standard error of the mean (SEM) is indicated on the bar.

Throughout the experiment, the number of trapped insects increased in each section in both groups (L and Bp). The orthogonal contrast analysis revealed no significant differences in the number of insect pests in traps placed in different sections in group L (C#1 *p* = 0.3718; C#2 *p* = 0.8237). In group Bp, a significant difference was found between the average number of insect pests in traps placed in the middle section and the left and right section (C#1 *p* = 0.0030) in all trials (Table 3; Figure 2). 

In group L, the average number of insect pests was highest in traps No. 13, 4, 3, 14, and 12, and lowest in traps No. 6 and 1, which were placed in different sections of the house, and the repeatability of measurements was not observed. On days 10 and 20 of rearing, there were no significant differences in the number of insect pests between the traps in any of the trials. On days 31 and 40 of rearing, the number of insect pests in traps varied significantly in all trials. No repeatable “critical points” were recorded in group L. In group Bp, the average number of insect pests was highest in traps No. 8, 9, and 7 placed in the middle section (“critical points”) and lowest in traps No. 15, 2, 6, 1, 5, and 11. In this poultry house, the number of pests in traps was significantly different already on day 10 of rearing, and it was repeatable in each trial. The Student’s *t*-test revealed that on day 10 of rearing, there was no significant difference in the number of pests located in the “critical points” between the groups (*p* = 0.1562). On the remaining days of measurement, the average number of pests was significantly higher in traps No. 7, 8, and 9 in group Bp than in group L (*p* < 0.001) (Table 4).

The results indicate that in group Bp, most insects accumulated at the “critical points” relative to the other traps, which was not the case in group L.

### 3.2. Microbiological Contamination

The total counts of mesophilic aerobes and fungi in the air in groups L and Bp were compared based on of the overall mean per trial (trials I, II and III) (Table 5). As regards bacterial counts, the Student’s *t*-test revealed no significant differences between the groups in trials I and II, whereas a significant difference was found in trial III (*p* < 0.05). The counts of mesophilic aerobes were lower in group Bp. Similar results were obtained for fungal counts in the air. The Student’s *t*-test showed no significant differences between the groups in trials I and II, whereas a significant difference was found in trial III (*p* < 0.05). In trial II, the difference in fungal counts between the groups was at the limit of significance (*p* = 0.0660) (Table 5).

During the entire experiment, the average counts of mesophilic aerobes and fungi in the air in group L ranged from 1.14 to 3.17 log_10_ CFU/m^3^, and from 0.38 to 2.59 log_10_ CFU/m^3^, respectively. In group Bp, their ranges were as follows: mesophilic aerobes—1.12–2.92 log_10_ CFU/m^3^, fungi—0.18–2.12 log_10_ CFU/m^3^. A comparison of mean microbial concentrations on individual measurement days revealed significant differences in bacterial and fungal counts in air (*p* = 0.0001) and litter (*p* = 0.000007). In group L, the counts of mesophilic aerobes and fungi in the air increased during the rearing period. A different trend was observed in group Bp, where the counts of mesophilic aerobes and fungi decreased in some trials (Table 5).

The total counts of mesophilic aerobes and fungi in litter in groups L and Bp were compared based on the overall mean per trial (trial I, II, and III) (Table 6). The counts of mesophilic aerobes were significantly lower in group Bp in every trial (*p* < 0.05). In trial I, no significant differences in fungal counts were found between the groups (*p* = 0.2835). However, significant differences were noted in the other trials. Fungal counts in litter were significantly lower in group Bp (Table 6).

During the entire experiment, the average counts of mesophilic aerobes and fungi in litter in group L ranged from 7.47 to 10.64 log_10_ CFU/g and from 6.21 to 9.56 log_10_ CFU/g, respectively. In group Bp, their ranges were as follows: mesophilic bacteria—6.87–9.96 log_10_ CFU/g, fungi—5.19–8.94 log_10_ CFU/g. Microbial counts in litter increased in both groups throughout rearing. Moreover, in nearly all measurements, the biocidal paint was significantly more effective than liming—the counts of mesophilic aerobes and fungal counts in litter were higher in group L (Table 6). 

The results of a qualitative analysis of microorganisms in the houses are presented in Table 7 (air) and Table 8 (litter). The microflora identified in each group was quite differentiated and characterized by the presence of representatives of 15 genera/species in group L and 13 in group Bp. Similar microbial genera and species were identified in both groups during the experiment, with the predominance of *Proteus* spp., non-hemolytic *E. coli*, and *Enterobacter cloacae*. A qualitative analysis of litter revealed that fungi, *Cladosporium* spp. and *Candida krusei*, predominated in both groups. Moreover, *Aspergillus fumigatus* and *Penicillium* spp. were identified in group L, whereas *Rhodotorula* and *Trichosporon* were present in group Bp. *Salmonella* spp. were not detected in any of the measurements.

### 3.3. Microclimate Conditions and the Physicochemical Parameters of Litter

#### 3.3.1. Temperature, Relative Humidity, and NH_3_ Concentration in the Air

In this study, no significant differences in air temperature or relative humidity were found between the groups across trials. In both groups, the initial mean temperature was 34 °C ± 0.45 °C, and it decreased by 2 °C ± 0.55 °C each week. As a result, at the end of every rearing phase, the mean air temperature was 22 °C ± 0.81 °C in both groups (Table 9). The mean relative humidity ranged from 57% to 70% in both groups in each trial. Additionally, significant differences in air temperature were noted between the first and last day of rearing in groups L and Bp in each trial (Table 9).

On the first day of rearing, NH_3_ concentration was 0 ppm in both groups in each trial, and then it increased steadily. Significant differences in NH_3_ concentration in the air were noted in groups L and Bp on different days of rearing. In trial I, at the end of rearing, the mean NH_3_ concentration reached 19.20 ppm in group L and 14.87 ppm in group Bp; the respective values were 18.73 ppm and 11.60 ppm in trial II, 15.53 ppm and 12.67 ppm in trial III. On the last day of rearing, significant differences in NH_3_ concentration were found between the groups in every trial. The mean values were significantly higher in group L (Table 9). 

#### 3.3.2. Litter Temperature and Moisture Content, NH_3_ Concentration and pH

In trial I, litter temperature ranged from 16.87 °C to 23.02 °C and litter moisture content ranged from 9.47% to 38.20% in group L, whereas the respective values in group Bp were 19.65–26.68 °C and 9.07–41.60%. In trial II, the values of litter temperature and moisture were 16.65–23.68 °C and 9.20–36.60%, respectively, in group L and 17.00–23.25 °C and 9.13–40.00%, respectively, in group Bp. In trial III, the values of litter temperature and moisture reached 15.45–22.85 °C and 8.73–37.20%, respectively, in group L and 17.00–22.85 °C and 8.40–39.20%, respectively, in group Bp (Table 10). Significant differences in mean litter temperature were found between the groups in trial I. No significant differences in litter moisture content were noted between the groups across trials (Table 10). However, the values of this parameter increased during rearing.

Significant differences in NH_3_ concentration were observed between groups. Significantly higher values were recorded in group L, in the range of 0.27–32.60 ppm in trial I, 0.07–30.87 ppm in trial II, and 1.15–28.80 ppm in trial III. In contrast, in group Bp, the following ranges of values were noted: 0.07–23.47 ppm, 0.00–23.50 ppm, and 0.00–23.20 ppm in trials I, II, and III, respectively. In addition, the concentration of NH_3_ in litter increased throughout the rearing period in both groups, and such a trend was noted in all trials (Table 10).

No significant differences in litter pH were found between groups L and Bp in any of the trials or days of rearing (Table 10). In both groups, the pH values did not exceed 5–7 throughout the experiment.

## 4. Discussion

### 4.1. Location of A. diaperinus

Slow-release water-based insecticidal paints are becoming increasingly popular [53,54]. The application of insecticides inside houses can be an effective way of reducing insect pest populations by repelling, killing, or controlling infestations [55,56]. Many studies have investigated the effects of insecticidal paints on insect pests.

Amelotti et al. [57] evaluated the efficacy of an insecticidal paint based on organophosphate and pyrethroid formulations against *Triatoma infestans*. The researchers found that the pyrethroid and organophosphate formulations produced 84% and 98% mortality, respectively, after 12 months of application on different surfaces. Another study demonstrated that an insecticidal paint based on organophosphorus compounds was effective against mosquitos under both laboratory and field conditions. Mortality rates were 93–100% after 12 months of application in the laboratory [58]. However, the paint’s efficacy dropped to 60–80% after 12 months of application under field conditions [37]. Recent research has shown that an insecticidal paint formulation (active ingredient: deltamethrin, a synthetic pyrethroid) provided 18-month residual efficacy; 94% knockdown and 90% mortality of *Aedes aegypti* (a dengue vector) were achieved [54].

However, the effect of an insecticidal paint on *A. diaperinus* has not been evaluated to date. Dzik and Mituniewicz [29] reported that a biocidal paint was highly effective against the larvae and adults of *A. diaperinus* under laboratory conditions. However, its efficacy was considerably lower in production conditions—not all insect pests were killed. Nevertheless, the analyzed biocidal paint was more effective than the commonly used lime.

The present study was designed to determine whether the use of a biocidal paint as an insecticide and liming affect the location of *A. diaperinus* in traps in poultry houses. It was found that in group Bp, insect pests were located in the middle section of the house, especially in the “critical points”, i.e., the traps where the highest numbers of insects were collected (No. 7, 8, and 9). These traps were placed farthest away from the walls. This correlation was not observed in group L, which may indicate that the active ingredients of the insecticidal paint could influence insect locomotion. This observation may contribute to controlling *A. diaperinus* in poultry houses. If the places where insect pests are likely to be found in the highest numbers are known, additional pest management tools can be applied, including natural methods such as the use of essential oils. In a study by Wolf et al. [59], combined methods were most effective in controlling *A. diaperinus* populations. Therefore, the current study may provide a new path for research and contribute to reducing the use of chemicals in animal husbandry.

### 4.2. Microbiological Contamination

Microbiological contamination in poultry houses is higher than in other livestock buildings [60]. In order to assess the level of microbial contamination, the results of the present study were compared with the findings of other authors. It was found that the mean concentration of microbial contaminants in the air was lower than that reported by other researchers in both summer or winter seasons. In the present study, the average counts of mesophilic aerobes did not exceed 3.17 and 2.92 log_10_ CFU/m^3^, and average fungal counts did not exceed 2.59 and 2.12 log_10_ CFU/m^3^ in groups L and Bp, respectively. In the experiment performed by Mituniewicz et al. [23], different litter additives were used in three poultry houses. The average counts of microbial contaminants in these poultry houses ranged from 5.00 to 6.75 log_10_ CFU/m^3^ (bacteria) and from 5.01 to 6.50 log_10_ CFU/m^3^ (fungi) and were significantly higher than those obtained in this study. In the work of Wójcik et al. [25], the lowest mean bacterial and fungal counts were estimated at 5.00 log_10_ CFU/m^3^ and 4.55 log_10_ CFU/m^3^, respectively, in the summer season. The respective values determined in the winter season were even higher. Higher values were also reported by Lawniczek-Walczyk et al. [61]. In their study, the average levels of microbial (bacterial and fungal) air contamination during the initial sampling session were similar to those noted in the current study on the last day of rearing. Higher concentrations of airborne bacteria in poultry houses were obtained by Witkowska and Sowińska [21] and Kostadinova et al. [62]. Yang et al. [60] reported slightly lower concentrations of airborne bacteria and fungi than those determined in the cited studies, but they were still higher than in the present study (bacteria: 3.59–4.65 log_10_ CFU/m^3^, fungi: 2.37–3.68 log_10_ CFU/m^3^). In comparison with the findings of other authors, the values noted in the current experiment were much lower. Jiang et al. [63] tested various disinfectants, such as ozone, available chlorine, quaternary ammonium salt, glutaraldehyde, and mixed disinfectant. Bacterial aerosols were collected 72 h after disinfection of broiler houses. In the case of available chlorine, the total bacterial count in the air was higher than in the present study at the beginning of bird rearing. The values noted for the remaining disinfectants were similar to or lower than those noted in the current study. These results may suggest that both liming and the analyzed biocidal paint were effective disinfection methods that improved the microbiological air quality in the poultry houses. However, significantly better results were achieved in group Bp.

The microbiological contamination of the litter in the poultry houses was also analyzed in relation to previous research. The mean total counts of mesophilic bacteria in litter, noted by Witkowska and Sowińska [21] after broiler houses had been fogged with aqueous solutions of peppermint oil and thyme oil, were similar to those obtained in the current study in group Bp. The respective values in group L were slightly higher. At the end of week 3 of rearing, the mean bacterial load in the litter was close to or exceeded 10.00 log_10_ CFU/g. The maximum microbial load in litter reported by Kostadinova et al. [62] was lower than the values obtained in the present study. Milanov et al. [64] assessed the total counts of aerobic microorganisms (including aerobic mesophilic bacteria, yeasts, and molds) in two poultry houses, which were found to be lower than the counts of mesophilic aerobes and fungi in litter, determined in the present study. Viegas et al. [65], Witkowska et al. [66] and Ostović et al. [67] also reported lower levels of fungal contamination in litter, compared with the present findings. Thus, it appears that neither liming nor biocidal paints are as effective in reducing the microbial load in litter as disinfectants in the form of, e.g., litter additives. However, the tested biocidal paint is more effective than liming. A combined method involving the application of a biocidal paint followed by a disinfectant (e.g., natural essential oils) added to litter could be considered.

It is widely acknowledged that microbial concentrations in the air and litter increase in poultry houses during the production cycle [19,21,23,68]. The results obtained in the current study varied over wide ranges. Nevertheless, the concentration of mesophilic aerobes in the air was generally higher than the concentration of airborne fungi, which is consistent with the findings of Wójcik et al. [25], Yang et al. [60], and Lawniczek-Walczyk et al. [61].

*Salmonella* is one of the major pathogens in the poultry industry because it is responsible for poultry-to-human transmission and economic losses [69,70]. As an EU Member State, Poland must annually report the results of the National Control Program for *Salmonella* in broiler flocks [16] in order to reduce the prevalence of foodborne zoonotic agents and the risk they pose to public health [71]. The recommended measures include preventing the introduction of *Salmonella* onto chicken farms and its on-farm transmission [70]. Thus, it is important to maintain the highest standards of hygiene on the farm. Furthermore, effective disinfectants must be applied due to the increasing antimicrobial resistance of *Salmonella* spp. The level of *Salmonella* resistance varies from country to country and depends on many factors [72]. The emergence of antimicrobial resistance in poultry production is a serious public health concern [73]. Therefore, *Salmonella* spp. should be effectively controlled in poultry houses. In the present study, the pathogen was not detected in either poultry house across trials.

Indoor contamination, including microbiological contamination, in intensive poultry farming poses a serious threat to bird and human health [60,74]. The microorganisms identified in the air and in litter are most commonly encountered in poultry facilities and have also been reported by other researchers [21,23,25,60,63,67,68,74]. Mesophilic aerobes detected in the air and fungi identified in litter were similar in both groups. However, pathogens such as *Klebsiella pneumoniae* and *Enterobacter faecalis* were not found in group Bp, which is good because *K. pneumoniae* is the most medically important species of *Klebsiella* spp. In humans, it can cause respiratory and urinary tract infections. In poultry, *K. pneumoniae* is one of the respiratory pathogens responsible for high mortality rates in chicks and adult birds [75]. In addition, *K. pneumoniae* isolated from poultry farms has been reported to acquire increasing levels of antimicrobial resistance [76]. In turn, *E. faecalis* is one of six species of the genus *Enterococcus* associated with poultry diseases, which affects avian species of all ages [77]. Bacteria that rapidly develop resistance to multiple antimicrobials are particularly dangerous. *Enterobacter faecalis* from broiler breeders has been implicated in its vertical transmission to their offspring [78]. In contrast, mycotoxin-producing *Penicillium* spp. and *Aspergillus fumigatus*, which cause penicilliosis and aspergillosis, respectively, were not detected in litter in group Bp in the present study, although these fungi are frequently found in litter in broiler houses [66,79,80,81]. Furthermore, *Alternaria* fungi, which have been identified in poultry litter by many researchers, were not detected in the analyzed facilities, either.

These results may suggest that the tested biocidal paint improves the microbiological quality of air and litter and that it is more effective than liming.

### 4.3. Microclimate Conditions and the Physicochemical Parameters of Litter

The counts of airborne microorganisms in livestock buildings are determined by numerous factors such as temperature, humidity, air velocity inside the building, efficiency and type of ventilation, NH_3_ concentration, and litter quality [23,24,25]. These parameters affect the level of hygiene in poultry houses.

The microclimate conditions in the analyzed poultry houses were comparable with those reported by other researchers [18,23,25,61,66,81,82,83,84]. Indoor air temperature was highest in the first week of rearing, and then it decreased significantly in both groups in all trials; relative humidity remained at a similar level during rearing in both groups in all trials; NH_3_ concentration increased steadily during the experiment. The tested biocidal paint had no significant effect on microclimate conditions.

In intensive broiler production systems, stocking density in poultry houses is very high, which makes it difficult to maintain optimal microclimate and hygiene conditions. One of the reasons is that manure and bedding remain inside poultry houses during the entire production cycle. Poultry litter is a mixture of litter material, feed, manure, feathers, and other detritus particles, which can be a source of environmental contamination, posing a health risk to broilers [23,27,62,80,82]. Poor litter quality promotes fungal growth; therefore, litter should be continuously monitored, mainly by assessing its moisture content [67,79], NH_3_ concentration [23], and pH [22,27]. However, according to Arné et al. [79], the effect of changing litter moisture and pH on fungal population densities remains controversial, although damp and dirty sites may enhance fungal growth. The physicochemical parameters of litter, noted in this study, are similar to those reported by Mituniewicz et al. [27], Ostović et al. [67], and Iwańczuk-Czernik et al. [82]. These results may indicate that both liming and the analyzed biocidal paint provide appropriate litter hygiene. However, litter moisture content and NH_3_ concentration were significantly more favorable in group Bp.

In this study, the microclimate conditions in both poultry houses and the physicochemical parameters of the litter were consistent with the current EU [85] and Polish [86] legislation and with the relevant recommendations [67,87,88].

## 5. Conclusions

The results of this study indicate that *A. diaperinus* were found in traps placed farther away from the walls of the analyzed poultry houses due to the repellent properties of the tested biocidal paint. An analysis of the number of insect pests found in traps placed in different sections of the house and the identification of “critical points” could have important practical implications, paving the way for future research aimed at controlling insect pest populations. Furthermore, the slow-release water-based biocidal paint, tested in this experiment, seems to be a more effective disinfection method than liming, which could contribute to reducing the incidence of infectious diseases in poultry flocks. The present and previous research findings suggest that the tested biocidal paint could be an effective alternative to other disinfectants. No significant differences were noted between the biocidal paint and liming in terms of their effects on the microclimate conditions in the analyzed poultry houses. However, the application of the biocidal paint decreased the litter moisture content and NH_3_ concentration to a greater extent than liming.

Further research is needed. Perhaps a combination method would prove effective: applying natural antimicrobial and insecticidal methods with a simultaneous reduction in chemical could prevent the growing resistance of the microorganism and insects to the active substances.

## Figures and Tables

**Table 1:** Experimental design.

Trial	Number of Production Cycles over 423 Days	Insect Collection (Day of Rearing)	Date of Treatment against *A. diaperinus*	
			Liming (L)	Biocidal Paint (Bp)
I	1st	10	7 days before the 1st production cycle	7 days before the 1st production cycle
		20		
		31		
		40		
II	3rd	10	7 days before the 3rd production cycle	
		20		
		31		
		40		
III	7th	10	7 days before the 7th production cycle	
		20		
		31		
		40		

**Table 2:** Set of orthogonal contrasts.

Contrast	Section		
	Left	Middle	Right
C#1 middle vs. left and right	−1	2	−1
C#2 left vs. right	1	0	−1

**Table 3:** Section-by-section analysis of orthogonal contrasts.

Contrast	Section			*p*-Value
	Left	Middle	Right	
C#1 middle vs. left and right	−1	2	−1	
Liming				0.3718
Biocidal paint				0.0030
C#2 left vs. right	1	0	−1	
Liming				0.8237
Biocidal paint				0.6033

**Table 4:** Logarithmic mean of the number of pests in the “critical points”.

Day of Rearing	Log Mean L	Log Mean Bp	*p*
10	0.58	0.88	0.1562
20	1.48	2.40	0.0008
31	1.62	2.73	0.0001
40	2.33	2.87	0.0004

Student’s *t*-test was used to compare L (Liming) and Bp (Biocidal paint) groups; significant differences with *p* < 0.05.

**Table 5:** Mean values of the total counts of mesophilic aerobes and fungi in the air [log_10_ CFU/m^3^].

		Liming (L)				Biocidal Paint (Bp)				*p*-Value L vs. Bp
		Day of Rearing				Day of Rearing				
		1	21	40	${}^{1}\bar{x}\;\pm \;\mathrm{SEM}$	1	21	40	${}^{1}\bar{x}\;\pm \;\mathrm{SEM}$	
Mesophilic aerobes	Trial I	1.14	2.64	2.40	2.06 ± 0.11	2.69	2.50	1.12	2.11 ± 0.11	0.7058
	Trial II	2.85	2.21	3.17	2.74 ± 0.06	2.49	2.37	2.92	2.59 ± 0.06	0.2494
	Trial III	2.54	2.91	3.09	2.85 ± 0.08	2.34	2.61	2.80	2.58 ± 0.08	0.0462
Fungi	Trial I	0.38	1.70	1.99	1.36 ± 0.09	2.03	1.46	0.18	1.22 ± 0.09	0.3355
	Trial II	2.59	1.41	2.02	2.01 ± 0.07	2.12	1.53	1.57	1.74 ± 0.07	0.0660
	Trial III	1.40	1.94	2.20	1.85 ± 0.12	1.30	1.32	1.61	1.41 ± 0.12	0.0044

^1^$\bar{x}\;\pm$ SEM, mean ± standard error of the mean; the total counts of mesophilic aerobes and fungi in the air were analyzed between L and Bp groups at 1, 21, and 40 days of rearing, respectively, in all trials followed by Student’s *t*-test, significant differences with *p* < 0.05.

**Table 6:** Mean values of the total counts of mesophilic aerobes and fungi in litter during the different rearing days in each trial [log_10_ CFU/g].

		Liming (L)				Biocidal Paint (Bp)				*p*-Value L vs. Bp
		Day of Rearing				Day of Rearing				
		1	21	40	${}^{1}\bar{x}\;\pm \;\mathrm{SEM}$	1	21	40	${}^{1}\bar{x}\;\pm \;\mathrm{SEM}$	
Mesophilic aerobes	Trial I	7.98	10.12	10.64	9.58 ± 0.18	7.84	9.17	9.96	8.99 ± 0.18	0.0121
	Trial II	7.47	9.24	10.22	8.98 ± 0.13	6.87	7.45	9.90	8.07 ± 0.13	0.0002
	Trial III	7.92	9.09	10.26	9.09 ± 0.16	7.06	7.96	9.77	8.26 ± 0.16	0.0006
Fungi	Trial I	6.21	7.91	9.56	7.89 ± 0.17	6.08	7.92	8.94	7.65 ± 0.17	0.2835
	Trial II	6.32	8.32	9.40	8.01 ± 0.18	5.19	6.77	8.19	6.72 ± 0.18	<0.0001
	Trial III	6.44	7.57	8.94	7.65 ± 0.13	5.49	6.86	8.35	6.90 ± 0.13	0.0019

^1^$\bar{x}\;\pm$ SEM, mean ± standard error of the mean; the total counts of mesophilic aerobes and fungi in the litter were analyzed between L and Bp groups on day 1, 21, and 40 of rearing, respectively, in all trials followed by Student’s *t*-test, significant differences with *p* < 0.05.

**Table 7:** Bacterial genera/species identified in the air.

	Liming (L)	Biocidal Paint (Bp)
Trial I	Non-hemolytic *E. coli*, *Brevundimonas diminuta*, *Enterobacter cloacae*, *Enterococcus faecalis*, *Klebsiella pneumoniae*, *Pantoea* spp., *Proteus mirabilis* *Proteus vulgaris* *Pseudomonas aeruginosa* *Staphylococcus saprophyticus*	Non-hemolytic *E. coli*, *Brevundimonas diminuta*, *Enterobacter cloacae*, *Micrococcus* spp., *Proteus vulgaris*, *Pseudomonas luteola*, *Pseudomonas aeruginosa*
Trial II	Non-hemolytic *E. coli*, *Enterobacter cloacae*, *Enterococcus faecalis*, *Klebsiella pneumoniae*, *Pantoea* spp., *Proteus* spp., *Staphylococcus saprophyticus*	Non-hemolytic *E. coli*, *Enterobacter cloacae*, *Pantoea* spp., *Proteus vulgaris* *Pseudomonas aeruginosa*, *Pseudomonas luteola*,
Trial III	Non-hemolytic *E. coli*, *Enterobacter cloacae* *Enterococcus faecalis*, *Klebsiella pneumoniae*, *Pantoea* spp., *Proteus* spp., *Staphylococcus saprophyticus*	Non-hemolytic *E. coli*, *Enterobacter cloacae*, *Pantoea* spp *Proteus* spp., *Pseudomonas aeruginosa*, *Staphylococcus saprophyticus*

**Table 8:** Bacterial genera/species identified in litter.

	Liming (L)	Biocidal Paint (Bp)
Trial I	isolated *Aspergillus fumigatus*, abundant growth with a predominance of *Cladosporium* spp., and numerous *Candida krusei*	isolated *Rhodotorula*, abundant growth with a predominance of *Cladosporium*, and numerous *Candida krusei*
Trial II	isolated *Candida krusei*, abundant growth with a predominance of *Aspergillus fumigatus*, and abundant growth of *Penicillium* spp.	isolated *Trichosporon, Candida krusei* and abundant growth with a predominance of *Cladosporium*
Trial III	isolated *Candida krusei* and *Penicillium* spp. as well as numerous *Aspergillus fumigatus*, and abundant growth with a predominance of *Cladosporium* spp.	isolated *Rhodotorula* and *Candida krusei* as well as abundant growth with a predominance of *Cladosporium* spp.

**Table 9:** Air microclimate parameters during the experiment.

Day of Rearing	Trial I						Trial II						Trial III					
	Liming (L)			Biocidal Paint (Bp)			Liming (L)			Biocidal Paint (Bp)			Liming (L)			Biocidal Paint (Bp)		
	Air temperature ( °C)	Relative humidity (%)	NH_3_ (ppm)	Air temperature ( °C)	Relative humidity (%)	NH_3_ (ppm)	Air temperature ( °C)	Relative humidity (%)	NH_3_ (ppm)	Air temperature ( °C)	Relative humidity (%)	NH_3_ (ppm)	Air temperature ( °C)	Relative humidity (%)	NH_3_ (ppm)	Air temperature ( °C)	Relative humidity (%)	NH_3_ (ppm)
1	34.00 ^A^ (±0.75)	67.30 (±7.56)	0.00 ^C^ (<0.001)	33.11 ^A^ (±0.68)	64.50 (±5.67)	0.00 ^C^ (<0.001)	33.96 ^A^ (±0.43)	63.00 (±5.43)	0.00 ^C^ (<0.001)	34.00 ^A^ (±0.55)	60.10 ^B^ (±6.11)	0.00 ^B^ (<0.001)	34.21 ^A^ (±0.51)	61.00 (±5.61)	0.00 ^C^ (<0.001)	34.45 ^A^ (±0.22)	59.00 (±4.58)	0.00 ^C^ (<0.001)
10	32.50 (±0.76)	60.50 ^B^ (±5.47)	1.13 ^C^ (±0.05)	32.10 (±0.31)	62.80 (±4.77)	1.20 ^C^ (±0.07)	32.11 (±0.46)	61.70 ^B^ (±2.91)	1.11 ^C^ (±0.10)	31.85 (±0.67)	62.20 (±4.45)	1.07 ^A^ (±0.06)	31.95 (±0.53)	60.60 (±2.76)	0.89 ^C^ (±0.11)	31.90 (±0.51)	57.50 ^B^ (±3.71)	0.73 ^C^ (±0.07)
20	29.95 ^AB^ (±0.45)	60.60 ^B^ (±5.01)	5.40 (±0.12)	30.55 (±0.29)	62.30 (±3.91)	2.47 ^C^ (±0.11)	29.91 (±0.11)	61.20 ^B^ (±3.33)	4.47 (±0.37)	29.85 ^AB^ (±0.41)	64.80 (±7.66)	3.40 (±0.15)	30.50 ^B^ (±0.74)	61.20 (±3.49)	5.27 ^B^ (±0.87)	30.00 ^B^ (±0.69)	65.50 (±4.92)	4.73 (±0.65)
31	28.50 (±1.12)	64.00 (±4.65)	15.33 ^B^ (±1.28)	28.40 ^B^ (±0.41)	64.30 (±4.76)	8.20 ^B^ (±1.43)	28.80 (±0.94)	67.00 (±6.21)	9.00 ^B^ (±1.15)	27.41 (±0.68)	69.00 ^A^ (±8.91)	7.80 ^AB^ (±1.03)	28.10 (±0.29)	62.90 (±5.28)	9.13 ^B^ (±3.45)	27.95 (±0.46)	66.10 ^A^ (±6.05)	8.73 ^B^ (±1.01)
40	26.11 ^B^ (±0.95)	68.00 ^A^ (±6.54)	19.20 ^aA^ (±5.34)	26.00 ^C^ (±0.33)	67.00 (±8.01)	14.87 ^bA^ (±4.48)	26.00 ^B^ (±0.27)	69.50 ^A^ (±4.98)	18.73 ^aA^ (±2.87)	26.00 ^B^ (±0.32)	69.10 ^A^ (±6.78)	11.60 ^bA^ (±2.89)	26.00 ^C^ (±0.46)	65.00 (±4.32)	15.53 ^aA^ (±4.68)	26.14 ^C^ (±0.48)	66.70 ^A^ (±5.40)	12.67 ^bA^ (±3.33)

± standard error of means (SEM); values followed by different lowercase letters in each line in each trial (differences in each parameter between groups L and Bp) are significantly different (*p* < 0.05) in the Student’s *t*-test, and values followed by different uppercase letters in each column are significantly different (*p* < 0.05) in Tukey’s multiple range test.

**Table 10:** Litter parameters during the experiment.

Day of rearing	Trial I								Trial II								Trial III							
	Liming (L)				Biocidal Paint (Bp)				Liming (L)				Biocidal Paint (Bp)				Liming (L)				Biocidal Paint (Bp)			
	Litter temperature ( °C)	Litter moisture (%)	NH_3_ (ppm)	pH	Litter temperature ( °C)	Litter moisture (%)	NH_3_ (ppm)	pH	Litter temperaturę ( °C)	Litter moisture (%)	NH_3_ (ppm)	pH	Litter temperaturę ( °C)	Litter moisture (%)	NH_3_ (ppm)	pH	Litter temperaturę ( °C)	Litter moisture (%)	NH_3_ (ppm)	pH	Litter temperaturę ( °C)	Litter moisture (%)	NH_3_ (ppm)	pH
1	16.87 ^bB^ (±5.65)	9.47 ^D^ (±2.51)	0.27 ^C^ (±0.02)	5.17 (±0.95)	19.65 ^aB^ (±6.14)	9.07 ^C^ (±1.77)	0.07 ^D^ (±0.01)	5.60 (±0.22)	16.65 ^C^ (±3.78)	9.20 ^B^ (±2.65)	0.07 ^C^ (±0.02)	5.71 (±0.35)	17.00 ^B^ (±7.32)	9.13 ^C^ (±2.50)	0.00 ^B^ (<0.001)	5.51 (±0.15)	15.45 (±4.22)	8.73 ^C^ (±4.39)	1.15 ^C^ (±0.30)	5.55 (±0.71)	17.00 ^B^ (±5.00)	8.40 ^C^ (±2.51)	0.00 ^C^ (<0.001)	5.34 (±0.18)
10	18.25 ^b^ (±5.05)	17.07 ^CD^ (±2.18)	2.40 ^a^ (±0.15)	5.20 (±0.41)	21.56 ^a^ (±4.67)	18.20 ^CB^ (±2.99)	0.40 ^bD^ (±0.08)	5.29 (±0.35)	17.63 (±5.60)	15.13 (±5.27)	3.60 (±0.47)	5.58 (±0.41)	19.20 (±6.29)	14.80 (±4.07)	0.53 ^B^ (±0.10)	5.49 (±0.37)	15.78 ^b^ (±5.91)	17.13 ^B^ (±3.91)	2.20 ^C^ (±0.25)	5.61 (±0.29)	18.15 ^a^ (±4.45)	16.87 (±4.35)	0.60 ^C^ (±0.05)	5.67 (±0.29)
20	20.05 ^bAB^ (±4.01)	21.51 ^C^ (±3.41)	7.07 ^a^ (±1.02)	5.24 (±0.22)	23.21 ^a^ (±3.90)	21.15 (±5.01)	3.40 ^bC^ (±1.00)	5.56 (±0.57)	17.95 ^b^ (±4.57)	20.80 (±4.78)	6.07 (±0.65)	5.63 (±0.39)	20.01 ^a^ (±6.88)	20.05 ^B^ (±3.32)	4.27 (±1.08)	5.65 (±0.71)	16.22 ^b^ (±5.63)	21.98 ^AB^ (±5.08)	7.00 ^BC^ (±3.78)	5.54 (±0.46)	18.93 ^a^ (±6.99)	20.17 ^B^ (±4.98)	4.27 (±1.95)	5.78 (±0.19)
31	21.15 ^b^ (±2.11)	29.88 ^B^ (±3.75)	20.73 ^aB^ (±4.80)	6.18 (±1.30)	24.07 ^a^ (±5.72)	31.87 ^B^ (±5.50)	15.93 ^bB^ (±2.63)	6.07 (±0.73)	18.92 ^B^ (±6.11)	27.65 ^AB^ (±7.02)	17.80 ^aB^ (±4.83)	5.66 (±0.58)	20.75 (±6.05)	30.01 ^AB^ (±6.09)	11.00 ^bAB^ (±4.23)	5.91 (±0.54)	18.95 (±7.20)	28.54 (±5.33)	18.40 ^aB^ (±5.29)	5.89 (±0.55)	20.05 ^A^ (±7.07)	26.30 ^B^ (±5.03)	10.06 ^bB^ (±4.82)	5.66 (±0.76)
40	23.02 ^bA^ (±3.19)	38.20 ^bA^ (±6.26)	32.60 ^aA^ (±7.33)	6.01 (±0.67)	26.68 ^aA^ (±2.05)	41.60 ^aA^ (±7.81)	23.47 ^bA^ (±5.06)	6.21 (±0.99)	23.68 ^A^ (±5.15)	36.60 ^bA^ (±6.81)	30.87 ^aA^ (±5.15)	6.01 (±0.63)	23.25 ^A^ (±5.95)	40.00 ^aA^ (±7.15)	23.50 ^bA^ (±4.41)	5.97 (±0.80)	22.85 (±6.73)	37.20 ^A^ (±8.10)	28.80 ^aA^ (±4.86)	6.03 (±0.42)	22.85 ^A^ (±6.23)	39.20 ^A^ (±6.65)	23.20 ^bA^ (±6.91)	6.12 (±0.82)

± standard error of means (SEM); values followed by different lowercase letters in each line in each trial (differences in each parameter between groups L and Bp) are significantly different (*p* < 0.05) in the Student’s *t*-test, and values followed by different uppercase letters in each column are significantly different (*p* < 0.05) according in Tukey’s multiple range test.

## Data Availability

The data presented in this study are available on request from the corresponding author.
